# Aesthetic Judgment in Calligraphic Tracing: The Dominant Role of Dynamic Features

**DOI:** 10.3390/bs15040525

**Published:** 2025-04-14

**Authors:** Qian Yuan, Guoying Yang, Ruimin Lyu

**Affiliations:** 1 School of Artificial Intelligence and Computer Science, Jiangnan University, Wuxi 214125, China; 6223110009@stu.jiangnan.edu.cn (Q.Y.); guoyingyang@jiangnan.edu.cn (G.Y.); 2 Jiangsu Key University Laboratory of Software and Media Technology under Human-Computer Cooperation, Jiangnan University, Wuxi 214125, China

**Keywords:** aesthetic judgment, dynamic features, static features, calligraphy tracing, aesthetic model, motion perception, experimental aesthetics, computational aesthetics

## Abstract

Aesthetic judgment in visual arts has traditionally focused on static features, yet research suggests that dynamic features also shape aesthetic experience. This study examines the dominance of dynamic features in calligraphic tracing aesthetics. Using a custom-designed calligraphy acquisition system, we recorded calligraphy experts and novices imitating Chinese characters and presented their works in three formats: static result sequence video *s*, pen-holding writing video *f*, and brushstroke trajectory video *b*. Participants then rated the stimuli on aesthetic dimensions. Results show that stimuli containing motion cues (*f* and *b*) received significantly higher ratings than static stimuli (*s*), confirming the positive role of dynamic features. Additionally, traced results maintained high structural similarity across writers. And the predictive power of static features for aesthetic scores was limited. This confirms the weak influence of static features on the aesthetics of calligraphic tracing. In conclusion, this study reveals that dynamic features play a dominant role in aesthetic judgment within the context of calligraphic tracing. These findings contribute to aesthetic modeling, proposing that observers dynamically adjust the weighting of static and dynamic features based on aesthetic context to form aesthetic judgments, thereby offering a novel perspective for research on aesthetic cognition mechanisms.

## 1. Introduction

Aesthetics is dedicated to investigating the nature of beauty and ugliness, human aesthetic awareness, aesthetic experience, and the underlying principles governing their creation and development J. Zhang et al. (2021). In contemporary society, aesthetic experience permeates various aspects of daily life, and with the advancement of science and technology, increasing research attention has been directed toward the multidimensional and complex nature of aesthetic judgment. In recent years, researchers have progressively recognized that aesthetic judgments of visual artworks are not solely dependent on their static visual features—such as symmetry, balance, and proportion—but may also be significantly influenced by dynamic features. These dynamic features refer to implicit cues within the aesthetic experience that evoke movement-related associations in observers. Such cues are often linked to the artist’s creative process or the observer’s perceptual experience, as exemplified by visible brushstrokes on a canvas or a seashore where you can stroll He et al. (2021); Sbriscia-Fioretti et al. (2013); Taylor et al. (2012). This discovery challenges the long-standing aesthetic theories predominantly centered on static features, prompting a critical reassessment: To what extent do static and dynamic features contribute to aesthetic perception? Could dynamic features potentially dominate aesthetic judgment? This question lies at the core of aesthetic cognition and holds profound implications for understanding the mechanisms of aesthetic judgment and the principles underlying aesthetic perception.

Historically, static features have long been regarded as key factors in explaining aesthetic experience Dziemidok (1993). As early as the 19th century, Gustav Fechner, the founder of experimental aesthetics, pioneered the idea that the aesthetic value of an artwork could be interpreted through the quantitative analysis of its objective formal characteristics Fechner (1876). This theory established a foundational link between static features and aesthetic perception, forming a crucial basis for subsequent research in visual arts. Static features can be categorized into two types based on extraction methods: manually designed static features Bhattacharya et al. (2010); Datta and Wang (2010); Su et al. (2011); Wu et al. (2010); Yeh and Cheng (2012) and deep learning-based static features Marchesotti et al. (2013); W. Wang et al. (2014); Chang et al. (2017); Jin et al. (2020); L. Zhang et al. (2014). Manually designed static features are further divided into low-level static features and high-level static features Meng et al. (2023). Low-level static features describe the fundamental attributes of visual artworks, such as color, edge distribution, and texture. These features are directly extracted from the external form of an input image and are widely used in aesthetic assessments of paintings Aleem et al. (2017); C. Li and Chen (2009); McManus et al. (1985) and photographs Jahanian et al. (2015); Ke et al. (2006); Y. Zhou et al. (2014). High-level static features, on the other hand, characterize the content attributes of artworks and are employed to investigate the impact of semantic information on aesthetic evaluation in visual art Dhar et al. (2011); Iigaya et al. (2021); Obrador and Moroney (2009). In the fields of experimental aesthetics and computational aesthetics, researchers have designed aesthetic experiments to validate the relationship between static features and the aesthetic process. Dotta et al., through an aesthetic discrimination experiment ranking 56 static features, identified that mean hue, average pixel intensity, and they found that a saturation-based rule of thirds measure contributed the most to an image’s aesthetic value Datta et al. (2006). Similarly, Mallon et al. examined aesthetic perception in abstract paintings and found that static features related to color properties and self-similarity significantly influenced aesthetic evaluation Mallon et al. (2014). In addition, several manually designed static features, such as brightness (color balance) Aragón et al. (2008); Graham et al. (2016); Pinto et al. (2006), complexity Berlyne (1974); Bies et al. (2016); Rigau et al. (2008), and symmetry McManus et al. (2011); Westphal-Fitch et al. (2013), have been confirmed to be highly correlated with the aesthetic process. With the interdisciplinary integration of computer vision and artificial intelligence, research on aesthetic processes has evolved from manually designed static features to deep learning-based static features. A common approach involves using neural network models to automatically extract static features from visual aesthetic works and develop aesthetic evaluation models, thereby simulating human aesthetic judgment through computational methods. This line of research has further confirmed the significant impact of deep static features on aesthetic judgment. For example, Lu et al. proposed an image aesthetic evaluation model based on a dual-column deep neural network, which simultaneously extracts global and local deep static features, thereby constructing a more accurate framework for visual aesthetic assessment Lu et al. (2015). While these studies provide essential methodological support for understanding aesthetic perception, they primarily focus on the independent role of static features, largely neglecting the potential influence of dynamic features.

In recent years, research in cognitive psychology and neuroscience has provided growing evidence that, in addition to static features, dynamic features also play a crucial role in the aesthetic perception of visual art. Studies in neuroaesthetics have revealed that when observers engage in aesthetic perception of visual artworks, the brain regions associated with motion processing exhibit significant activation. This process involves the mirror neuron system simulating motion-related information Bara et al. (2021); Freedberg and Gallese (2007); Thakral et al. (2012), offering physiological evidence for the role of dynamic features in aesthetic experience. Neuroscientific approaches have confirmed that dynamic features are inherently present in visual artworks. On the one hand, direct dynamic features, which contain explicit motion-related information such as direction and speed, contribute to the enhancement or even creation of motion perception. These include motion lines, stroboscopic effects, and blur Krekelberg et al. (2005); Pavan et al. (2011). On the other hand, indirect dynamic features, which are associated with the observer’s personal cognition, can also evoke motion-related associations. For example, when calligraphy learners appreciate a calligraphic work, they may unconsciously simulate the process of calligraphy creation based on their prior experience—an instance of motion association triggered by dynamic features. Research on the relationship between dynamic features and aesthetic judgment has largely been conducted within the frameworks of experimental aesthetics and neuroaesthetics. For instance, Ticini et al. found through experimental studies that when observers perceived gesture-based motion cues that aligned with the artist’s creative style, their aesthetic preference ratings significantly increased Ticini et al. (2014). Similarly, research by Williams et al. demonstrated that motion stimuli consistent with the artist’s creative traces significantly prolonged observers’ gaze duration, thereby enhancing aesthetic experience Williams et al. (2020). These findings underscore the importance of dynamic features in aesthetic judgment, suggesting that, like static features, dynamic features possess explanatory power in shaping aesthetic experience.

**Figure 1:**
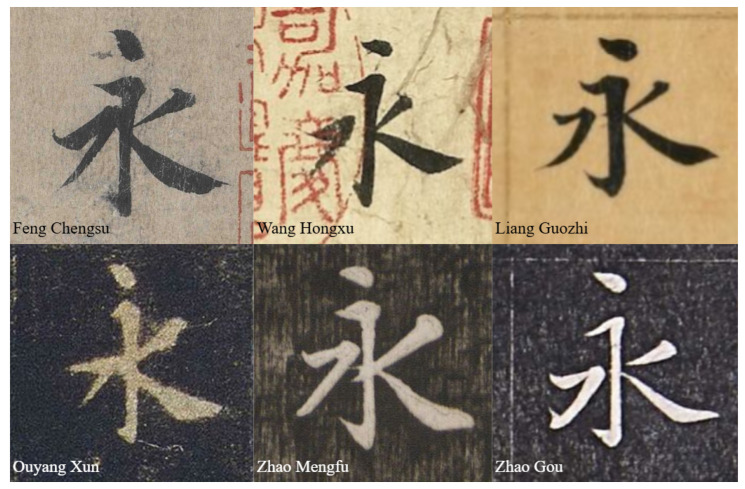
This figure presents a comparison of the character Yong in different traced copies of *Lanting Xu*. This raises a critical question: Why do traced works with highly similar forms evoke distinctly different aesthetic experiences? Could this difference be attributed to implicit dynamic features?

Most models of aesthetic experience include three fundamental stages: the perceptual stage, cognitive stage, and emotional stage Cupchik and László (1992); Höfel and Jacobsen (2007); Leder et al. (2004). After recognizing the significance of implicit dynamic features, researchers have attempted to explain the presence of them by examining the interactive processing across multiple stages within aesthetic models Di Dio et al. (2016). However, the relative weighting of static and dynamic features in aesthetic judgment remains underexplored, and existing aesthetic model frameworks fail to provide a comprehensive explanation for this issue. Moreover, as highlighted in previous analyses, most traditional aesthetic research has predominantly focused on static features. This raises a critical question: Are there specific aesthetic contexts in which dynamic features can surpass static features and dominate aesthetic judgment? A compelling case for investigating this question lies in Chinese calligraphy. Among various art forms, calligraphy exhibits unique aesthetic properties, as it not only showcases static features such as stroke forms and composition rules but also conveys dynamic features through brushstroke traces, which encode variations in speed, pressure, and directional transitions during the creation process Xu et al. (2016). Thus, calligraphy serves as an ideal medium for exploring the dominance of dynamic features in aesthetic perception. Additionally, in the process of learning calligraphy, there are two distinct training methods: the observational method and the tracing method. Specifically, the observational method involves placing the calligraphy model sheet beside the learner, who carefully observes the character’s structure and brush techniques before independently completing the writing. In contrast, the tracing method requires overlaying a thin transparent sheet directly on top of the model sheet, allowing the learner to trace each stroke precisely and ensuring a high degree of structural similarity in the reproduced character. Given these distinctions, the tracing method is more suitable for the experimental design of this study, as it effectively minimizes interference from static features while highlighting the implicit dynamic features present in the writing process. Additionally, in the context of the tracing method, learners acquire writing techniques by replicating the static structure of an original masterpiece. However, due to individual writing habits, their dynamic execution during reproduction often deviates significantly from the original work, potentially leading to variations in aesthetic experience. A notable example is the classic calligraphy masterpiece Lanting Xu, of which multiple traced copies by different calligraphers exist. Despite their high structural similarity due to the tracing method, observers still exhibit considerable differences in their aesthetic appreciation of these works Z. Chen (2012). Figure 1 illustrates several traced versions of the character Yong from *Lanting Xu*. While their forms appear nearly identical, they evoke distinct aesthetic experiences. Based on this observation, we hypothesize that in the aesthetic context of calligraphic tracing, dynamic features may exert a dominant influence on aesthetic judgment.

Building on the aforementioned background, this study seeks to address the following key research questions: To what extent do static and dynamic features contribute to aesthetic perception? Furthermore, are there specific aesthetic contexts in which dynamic features can surpass static features and dominate aesthetic judgment? To explore these questions, we propose the core hypothesis: “In the aesthetic context of calligraphic tracing, dynamic features dominate aesthetic judgment”. This hypothesis is further divided into two testable subhypotheses:

**Hypothesis** **1.** *Dynamic features have a significant influence on aesthetic judgment.*

**Hypothesis** **2.** *The impact of static features on aesthetic judgment is relatively weak.*

**Figure 2:**
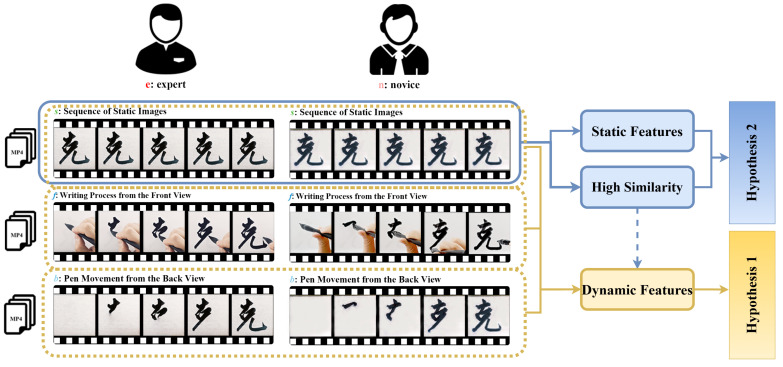
This figure shows the experimental framework for evaluating the aesthetic impact of static and dynamic features in calligraphic tracing.

This study adopts a combined approach of experimental aesthetics and computational aesthetics. The experimental framework is shown in Figure 2. We invite both expert and novice calligraphers to replicate representative character samples from classic calligraphic works using the tracing method. The aesthetic evaluation of the traced results is conducted based on three different presentation formats—static result sequence video *s*, pen-holding writing video *f*, and brushstroke trajectory video *b*—leading to the establishment of an aesthetic evaluation dataset for calligraphic tracing. On the one hand, by analyzing aesthetic rating variations across different presentation formats, we aim to determine whether the addition of dynamic features significantly influences aesthetic judgment, thus testing Hypothesis 1. On the other hand, we examine the role of static features in the aesthetic perception of calligraphic tracing from two perspectives: First, we conduct a quantitative analysis of structural similarity to assess the extent of static form variations in traced results. Second, based on these structural variations, we further evaluate the predictive power of classic static features. Specifically, we extract 26 classical static features from the traced results and progressively analyze their predictive capability by constructing multiple linear regression models and neural network models, thereby testing Hypothesis 2. If the experimental findings support these two hypotheses, this would provide empirical evidence that in the aesthetic context of calligraphic tracing, implicit dynamic features play a dominant role in aesthetic judgment.

Based on the experimental findings, this study further proposes a dynamically adaptive aesthetic model. The core concept of this model is that during the aesthetic process, observers do not rely solely on either static or dynamic features in isolation. Instead, they dynamically adjust the weighting of these features based on factors such as the type of artistic material, the context of creation, and the cultural setting, thereby influencing aesthetic judgment. For instance, in the aesthetic context of calligraphic tracing, dynamic features tend to play a dominant role, whereas in other forms of visual art, such as photography, static features may be more critical. This adaptive mechanism helps explain why certain classical aesthetic features fail to reproduce consistent aesthetic effects across different types of visual artworks. The construction of this model not only offers a new perspective on the dynamic features of aesthetic mechanisms but also lays a solid theoretical foundation for the refinement and application of aesthetic models in a broader context.

## 2. Materials and Methods

### 2.1. Participants

A total of 198 undergraduate students from Jiangnan University, majoring in Digital Media Technology and Artificial Intelligence, participated in the aesthetic evaluation experiment of this study. The mean age of the participants was 21.4 years (SD = 1.4), comprising 72 women and 126 men. The majority of participants were right-handed (189 individuals), while a small proportion were left-handed (9 individuals). All participants had normal or corrected-to-normal vision and were native Mandarin speakers to ensure their ability to accurately comprehend the calligraphic aesthetic evaluation tasks and provide reliable feedback during the experiment.

### 2.2. Stimuli

The stimuli used in this study were selected from a self-constructed calligraphic tracing video dataset. This dataset consists of recorded videos of a highly experienced calligraphy expert (e) and a novice (n) replicating Chinese characters within a customized calligraphy acquisition system. To ensure that the selected character samples were both representative and widely applicable, three key dimensions were considered: character structure (six distinct structural types), complexity (high, low), and calligraphic style (Yan Zhenqing, Liu Gongquan, Ouyang Xiu, Zhao Mengfu). A total of 48 character samples were selected according to these criteria. The chosen Chinese characters, along with the static traced results produced by the calligraphy expert and the novice, are presented in Table 1.

**Figure 3:**
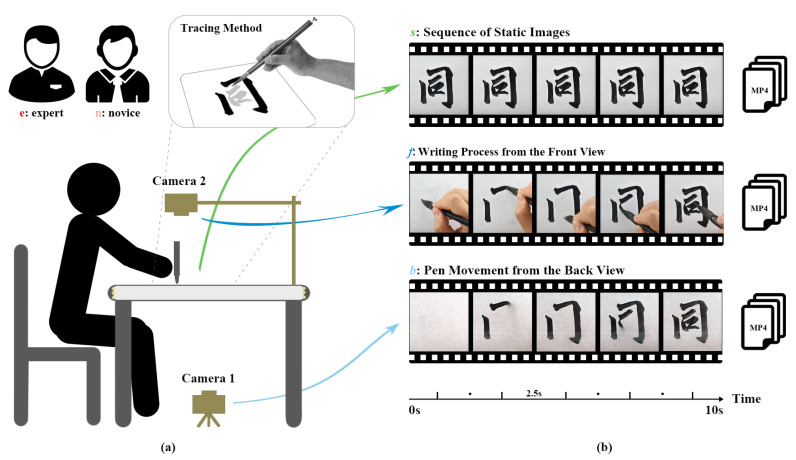
Overview of the calligraphy acquisition system and video data formats. (**a**) Schematic diagram of the calligraphy acquisition system. (**b**) Examples of three video data formats: static result sequence video *s*, pen-holding writing video *f*, and brushstroke trajectory video *b*.

During the video acquisition process, we invited a highly experienced calligraphy expert and a novice learner to imitate all character samples using a custom-designed calligraphy acquisition system. Prior to the experiment, both participants received detailed written and verbal instructions and provided written informed consent. The design of the calligraphy acquisition system is illustrated in Figure 3a, which includes two cameras: Camera 1, mounted beneath a glass tabletop, captures the motion trajectory of brushstrokes on the paper from a bottom-up perspective; Camera 2, positioned above the table, records the full-handwriting process, including hand movements, from a top-down perspective. The acquisition system ultimately generates three types of video data, as shown in Figure 3b: static result sequence video *s*, a sequence of frames representing the final written outcome; pen-holding writing video *f*, captured from a frontal perspective showing the pen-holding and writing movement; and brushstroke trajectory video *b*, recorded from a rear perspective and focusing on brush motion on the paper.

To maintain video quality and consistency, all videos in the dataset adhere to the same technical specifications, including a fixed aspect ratio (1:1), a resolution of 960 × 960 pixels, and a standardized video compression format (H.264). These specifications ensure clear visualization of writing details, providing reliable data support for subsequent aesthetic evaluation tasks.

### 2.3. Procedure

The stimuli in the dataset were classified into four groups based on character structure, complexity, and calligraphic style. Each group contained 12 characters, with each character presented in three formats (static result sequence video *s*, pen-holding writing video *f*, and brushstroke trajectory video *b*), resulting in 36 stimuli per group (12 characters × 3 formats) and a total of 144 stimuli across all four groups. Participants were randomly assigned to one of the groups to ensure exposure to different stimulus combinations while effectively controlling for inter-group interference and bias. This design also ensured data diversity and broad applicability.

Before the experiment, all participants received detailed written and verbal instructions to ensure a full understanding of the experimental procedure, rating criteria, and key considerations. To minimize visual distractions, participants were required to set their browsers to full-screen mode and were instructed to avoid any external interruptions (e.g., phone calls, notifications, etc.) during the test. The entire experiment was designed to be completed in a single session of approximately 30 min to prevent attention loss or fatigue effects due to prolonged testing.

**Figure 4:**
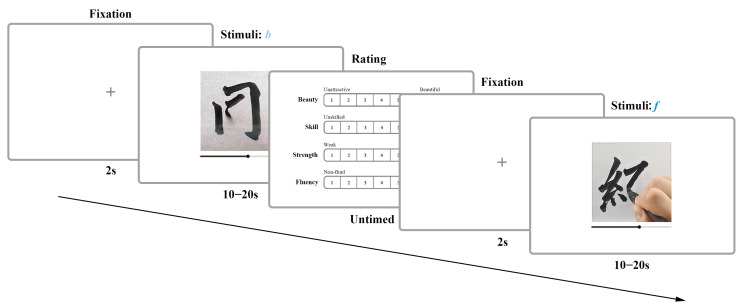
This figure shows the complete process of the aesthetic evaluation experiment: after a 2-s fixation interface, the participant proceeds to the stimulus display interface, where they watch the video. Then, the rating phase begins (Rating, no time limit), where participants evaluate the stimulus on a seven-point scale across four dimensions (beauty, skill, strength, fluency). After completing the rating, they return to the fixation interface, and the cycle repeats for the next stimulus.

During the formal experiment, participants viewed each video stimulus in a randomized order and rated it using a seven-point scale, as shown in Figure 4. The rating scale ranged from 1 (most negative aesthetic evaluation) to 7 (most positive aesthetic evaluation) and covered four evaluation dimensions: beauty, skill, strength, and fluency. The selection of rating dimensions was based on both general aesthetic perception and key subjective motion perception metrics. In addition to the universal aesthetic dimension “beauty”, we incorporated representative dimensions for assessing subjective motion perception, referencing traditional Chinese calligraphy evaluation systems and contemporary cognitive science theories. Among these, strength was selected as a fundamental attribute of motion perception, while fluency was included due to its recognized impact on aesthetics—previous research has shown that fluent motion is often perceived as more aesthetically pleasing Hogan and Flash (1987). Additionally, skill, a key measure of calligraphic proficiency, was incorporated as an aesthetic evaluation dimension. While these four dimensions do not fully capture the complexity of calligraphic aesthetics, they expand the evaluation perspective by emphasizing subjective motion perception in aesthetic judgment. Ultimately, the experiment generated the aesthetic evaluation dataset, which contains ratings across four dimensions, reflecting the interplay between motion perception and aesthetic experience in calligraphic tracing. This dataset provides multidimensional empirical support for examining the respective roles of dynamic and static features in aesthetic judgment.

### 2.4. Analysis 1: The Influence of Dynamic Features on the Aesthetic Evaluation of Calligraphic Tracing

To examine the positive impact of dynamic features on the aesthetic perception of calligraphic tracing, this section employs one-way analysis of variance to assess the significance of differences in four aesthetic dimensions (strength, fluency, beauty, and skill) across three different presentation formats of calligraphic tracing (static result sequence video *s*, pen-holding writing video *f*, and brushstroke trajectory video *b*). ANOVA is a widely used statistical analysis method for comparing whether the means of multiple sample groups differ significantly. The fundamental principle of ANOVA involves variance decomposition, where the total variability is divided into within-group variance and between-group variance, followed by the calculation of the *F*-statistic. If the *F*-statistic is significantly greater than the critical value at the predefined significance level, the null hypothesis is rejected, indicating a significant difference between groups. Conversely, if the *F*-statistic does not exceed the critical threshold, the null hypothesis cannot be rejected, implying that the group means are not significantly different.

Using this approach, we can analyze how different presentation formats of calligraphic tracing influence aesthetic ratings and evaluate whether dynamic features significantly enhance aesthetic evaluation. The core objective of this analysis is to quantify the independent contribution of dynamic features to aesthetic judgment and to test the validity of the “dynamic features dominate” hypothesis in the calligraphic tracing aesthetic context. The data analysis procedures are as follows:

1. Descriptive statistics of rating data: Conduct descriptive statistical analysis to obtain mean values, standard deviations, and other fundamental statistics for each presentation format across different aesthetic dimensions, providing a basis for subsequent analyses.
2. The role of motion information in aesthetic evaluation: To investigate the impact of dynamic features on aesthetic evaluation, one-way ANOVA is performed to test whether the presentation format (*s*, *b*, *f*) has a significant effect on the four aesthetic dimensions (beauty, fluency, strength, and skill). Through this analysis, we can determine whether different presentation formats exert an independent influence on aesthetic judgment.

All data analyses were conducted using SPSS 16.0.0, employing descriptive statistics and one-way ANOVA, with a significance level set at *p* < 0.001. The analysis aims to validate the positive influence of dynamic features in the aesthetic perception of calligraphic tracing.

### 2.5. Analysis 2: The Influence of Static Features on the Aesthetic Evaluation of Calligraphic Tracing

After examining the impact of dynamic features on the aesthetic perception of calligraphic tracing, it is essential to further analyze the role of static features in this context. This section investigates two key aspects: (1) differences in the static form of traced results between different writers and (2) the predictive power of static features in aesthetic evaluation.

#### 2.5.1. Investigation of Morphological Differences in Traced Results

To assess morphological differences, we employed image similarity metrics to determine whether the static form of traced results differs significantly between calligraphy experts and novices. Specifically, we used three commonly applied image quality assessment metrics: PSNR (Peak Signal-to-Noise Ratio), which evaluates signal fidelity Hore and Ziou (2010); SSIM (Structural Similarity Index), which measures structural consistency Z. Wang et al. (2004); and FSIM (Feature Similarity Index), which assesses local feature matching accuracy Sara et al. (2019).These metrics comprehensively evaluate the degree of similarity in traced results across three dimensions—fidelity, structural coherence, and feature consistency—allowing us to assess the effectiveness of static morphological control. The data analysis procedure is as follows:

1. Baseline Consistency Test: We computed the similarity between the traced results of calligraphy experts (e)s and novices (n)s relative to the template group (s). Using PSNR, SSIM, and FSIM, we assesses static structural similarity between groups. This analysis aims to verify whether significant morphological differences exist between the expert and novice traced results.
2. Inter-group Similarity Analysis with Calligraphic-Style Variables: Building on the similarity analysis, we further introduced calligraphic style as a variable, categorizing the four styles: Yan Zhenqing, Liu Gongquan, Ouyang Xiu, and Zhao Mengfu. We assigned them id = 1, 2, 3, 4, respectively (a notation that will be used throughout the remainder of the study). By comparing similarities across different calligraphic styles and between different writers, we evaluated the degree of similarity among writers. A heatmap visualization was used to illustrate differences in similarity both across calligraphic styles and between individual writers.
3. Construction and Validation of Similarity Feature Ratios: To quantify the findings from step two and validate whether morphological differences between writers are effectively controlled, we constructed a similarity ratio Sa/Sb for analysis. Here, *Sa* represents the similarity of traced results between different writers, while *Sb* denotes the similarity between different calligraphic styles. By computing the Sa/Sb ratio, we can quantify the magnitude of morphological variation among writers.

All data analyses were conducted using SPSS 16.0.0, employing descriptive statistics, *t*-tests, and correlation analysis to systematically investigate morphological differences in traced results between different writers.

#### 2.5.2. Investigation of the Predictive Power of Static Features in Aesthetic Evaluation

To assess the predictive effectiveness of static features in aesthetic evaluation, we extracted 26 classical static features based on the morphological structure, ink distribution, and other artistic characteristics of calligraphy fonts to establish an aesthetic evaluation model for calligraphic tracing. These features capture aspects such as character complexity and contour properties (detailed in Section 3.2.2) and have been previously validated as effective descriptors of calligraphic aesthetics. The primary objective is to objectively evaluate their predictive capability in the aesthetic assessment of calligraphic tracing in this study.

For data analysis, we employed multiple linear regression and neural network models to model the relationship between static features and aesthetic ratings. The multiple linear regression model was used to analyze the linear relationship between static features and aesthetic scores, evaluating the explanatory power of each feature and its contribution to the ratings. Meanwhile, the neural network model was introduced to explore complex feature interactions and deeper feature patterns through nonlinear modeling, aiming to comprehensively capture the potential influence of static features on aesthetic evaluation in calligraphic tracing. The analysis procedure is as follows:

1. Multiple Linear Regression Analysis: Data analysis was conducted using SPSS 16.0.0, applying multiple linear regression to examine the linear relationship between static features and aesthetic ratings. The regression model was constructed by setting each aesthetic rating dimension (strength, fluency, beauty, and skill) as the dependent variable, while the static features (*f1*–*f26*) served as independent variables. The correlation between features and ratings was computed, and by analyzing regression coefficients and *R*^2^ values, we assessed the explanatory power and contribution of each static feature in predicting aesthetic ratings. However, since linear regression models may fail to capture the nonlinear relationships between static features and aesthetic ratings, we further introduced a neural network model.
2. Multilayer Neural Network Modeling Analysis: To comprehensively explore the predictive power of static feature combinations, we employed a multilayer perceptron (MLP) model to handle potential complex nonlinear relationships between classical static features and aesthetic ratings. Specifically, the input layer of the neural network consisted of 26 standardized static features, while the output layer corresponded to the four aesthetic rating dimensions (strength, fluency, beauty, and skill). The network was optimized by experimenting with multiple hidden layers and activation function combinations to identify the best model configuration for fitting the nonlinear patterns between static features and aesthetic ratings, thereby improving predictive accuracy. To prevent overfitting, we incorporated Dropout regularization and L2 regularization during training. Additionally, to accelerate convergence, we implemented the ReduceLROnPlateau callback function, which dynamically adjusts the learning rate during training. The neural network model was trained in a Python 3.11 environment.

Through the above statistical analyses, we systematically examined the predictive effectiveness of classical static features in the aesthetic evaluation of calligraphic tracing.

## 3. Results

### 3.1. The Influence of Dynamic Features on the Aesthetic Evaluation of Calligraphic Tracing

This section analyzes the impact of dynamic features on aesthetic judgment within the context of calligraphic tracing based on the evaluation data. The analytical approach follows two key steps: First, a descriptive statistical analysis of the rating data is conducted to obtain an overview of the scores, including fundamental statistics such as mean values and standard deviations across different presentation formats. Second, to investigate the role of dynamic features in aesthetic evaluation, an analysis of variance (ANOVA) is performed to determine whether presentation format significantly influences aesthetic ratings across different dimensions.

As shown in Table 2, the descriptive statistical analysis of the rating data reveals that the expert group demonstrated more consistent scoring, whereas the novice group exhibited greater variability, particularly in the static presentation condition. This result reflects the impact of expertise on aesthetic evaluation, suggesting that calligraphy experts, due to their experience, maintain a more stable level of assessment, whereas novices demonstrate greater fluctuations in their ratings due to their lack of expertise. Overall, the expert group consistently received higher scores across all aesthetic dimensions compared to the novice group. This preliminary finding suggests that, despite the high structural similarity in the static form of the traced results between experts and novices, participants were still able to differentiate the quality of the works based on additional “information”. We hypothesize that this additional “information” includes motion-related cues embedded in the writing results, a topic that will be further explored in subsequent discussions. Regardless of expertise level, participants assigned higher ratings in the two presentation formats containing direct motion information—namely, the brushstroke trajectory video *b* and pen-holding writing video *f*. This finding aligns with our prediction and further supports the hypothesis that dynamic features contribute significantly to aesthetic judgment in calligraphic tracing.

Next, to further investigate the impact of dynamic features on aesthetic ratings in calligraphic tracing, a one-way analysis of variance was conducted to examine significant differences in the four aesthetic dimensions across the three presentation formats (*s*, *b*, and *f*). The significance level for all statistical tests was set at *p* < 0.001. The results indicate that all four aesthetic dimensions exhibited highly significant between-group differences: strength (*F* = 19.089, *p* < 0.001), fluency (*F* = 25.052, *p* < 0.001), beauty (*F* = 10.848, *p* < 0.001), and skill (*F* = 18.710, *p* < 0.001). These findings demonstrate that presentation format significantly influences aesthetic evaluation across all dimensions.

**Figure 5:**
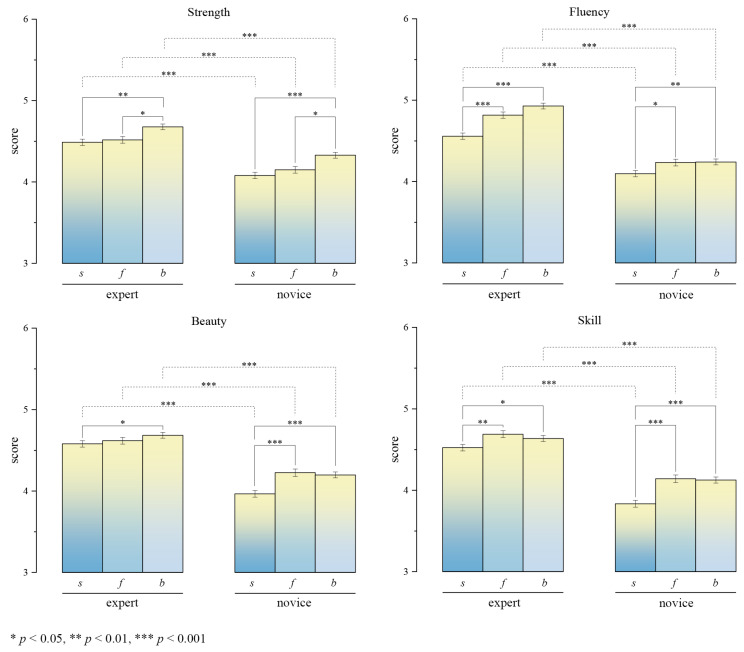
The figure shows the ratings of calligraphic works produced by experts and novices on three writing results (static result sequence video *s*, pen-holding writing video *f*, and brushstroke trajectory video *b*) in terms of strength, fluency, beauty, and skill. It can be found that the display method associated with motion information (*b*, *f*) can significantly improve aesthetic evaluation.

To further clarify the specific differences between groups, one-way ANOVA was separately conducted for the expert group and the novice group, followed by LSD post hoc tests. The results are presented in Figure 5. According to the ANOVA results, significant differences were observed in the static result *s* ratings between the expert group (e) and the novice group (n). We initially hypothesized that tracing method effectively controlled the static differences in traced results between different writers, ensuring high structural similarity between the expert and novice groups. However, despite this structural similarity, significant differences in aesthetic evaluation persisted between the two groups. This suggests that the static stimuli may contain implicit cues that lead to substantial variations in aesthetic judgment. To verify whether these “implicit cues” are associated with dynamic features, Section 3.2 will employ rigorous data analysis methods to examine the similarity of static morphology and the ineffectiveness of static features, thereby demonstrating the role of dynamic features even within static stimuli.

Returning to the analysis in this section, after completing the comparative analysis between different writers, we further conducted within-group analyses by examining aesthetic ratings for calligraphy experts and novices separately, focusing on the two presentation formats that contain direct motion cues (pen-holding writing video *f* and brushstroke trajectory video *b*).

In the expert group (e), the dynamic presentation formats (*b* and *f*) received significantly higher ratings than the static presentation format (*s*) in strength (*F* = 7.748, *p* < 0.001) and fluency (*F* = 27.042, *p* < 0.001). Although the skill dimension also exhibited a certain degree of significance (*F* = 4.310, *p* < 0.05), the overall difference was less pronounced compared to strength and fluency. Additionally, the difference in beauty (*F* = 2.010, *p* = 0.134) did not reach the significance threshold. Despite the fact that the differences in skill and beauty ratings did not achieve high statistical significance, the dynamic presentation formats (*b* and *f*) still exerted a positive influence on the ratings. Within the dynamic presentation formats, there were also notable differences in ratings between the pen-holding writing video *f* and the brushstroke trajectory video *b*. For the dimensions of strength, fluency, and beauty, the *f* condition received higher ratings than *b*, particularly in strength and fluency, which are closely related to motion perception. This suggests that, compared to the *b* condition, the *f* condition conveys more salient dynamic features. The enhanced perception of motion associated with the *f* condition appears to contribute to higher ratings in the beauty dimension.

For the novice group (n), all four dimensions, strength (*F* = 12.055, *p* < 0.001), fluency (*F* = 4.628, *p* < 0.001), beauty (*F* = 12.746, *p* < 0.001), and skill (*F* = 17.700, *p* < 0.001), showed significant between-group differences, indicating that presentation format had a substantial impact on the aesthetic ratings of the calligraphic works produced by novices. Notably, dynamic presentation formats (*b* and *f*) received significantly higher ratings across all dimensions compared to static presentation (*s*), demonstrating that the incorporation of direct motion information effectively enhances aesthetic evaluation, even more so than in the expert group. However, within the dynamic formats (*b* and *f*), further enhancement of dynamic features did not lead to higher ratings of the calligraphic works produced by novices. This is because the additional motion cues revealed more details about the novices’ actual skill, thereby limiting the extent of improvement in ratings.

Overall, these findings further confirm the positive role of dynamic features in enhancing aesthetic judgment in calligraphy, as dynamic features significantly improved the rating performance for both experts and novices. The effect was particularly prominent in the dimensions of fluency and strength, reinforcing the idea that motion cues play a critical role in shaping aesthetic perception in calligraphic tracing.

In summary, this study found that the presentation format of writing outcomes significantly influenced the aesthetic ratings of calligraphic works. The results indicate that the incorporation of direct motion cues enhances aesthetic evaluation across the four dimensions of strength, fluency, beauty, and skill, thereby confirming the positive role of dynamic features in the aesthetic perception of calligraphic tracing. These findings provide strong experimental evidence supporting Hypothesis 1, demonstrating the significant contribution of dynamic features to calligraphic aesthetic judgment.

### 3.2. The Weak Influence of Static Features on the Aesthetic Evaluation of Calligraphic Tracing

In the previous analysis, we confirmed the positive impact of dynamic features on the aesthetic judgment of calligraphic tracing and observed significant differences in ratings of static traced results between different writers. We hypothesize that, even when static forms are highly similar, observers can perceive differences in traced results through implicit motion cues. To test this hypothesis, this section focuses on the role of static features analyzed at two levels. First, we employed image similarity computation to verify whether morphological differences in traced results between different writers were effectively controlled. Second, based on the validated similarity in static form, we conducted quantitative analysis of the classical static features to assess their predictive effectiveness in calligraphic aesthetic evaluation. Specifically, we extracted 26 classical static features and utilized multiple linear regression and MLP neural network models to evaluate their predictive power in aesthetic ratings. Through this two-level analysis, we aim to clarify the role of static features in the aesthetic judgment of calligraphic tracing and further compare their influence relative to dynamic features.

#### 3.2.1. The Static Morphological Differences in Traced Results Are Not Significant

This study employed tracing method to rigorously regulate the morphological structure of traced results, minimizing static differences between different writers to the greatest extent possible. To systematically validate the static variations in writing outcomes, this section adopts a three-tier analysis based on similarity measures: (1) baseline consistency test for traced results between expert and novice groups; (2) inter-group similarity distribution analysis using calligraphic style as a reference variable; and (3) feature value construction for further quantitative validation. Three classical image similarity metrics were used to ensure a comprehensive evaluation of static morphological differences: PSNR (Peak Signal-to-Noise Ratio) for signal fidelity, SSIM (Structural Similarity Index) for structural consistency, and FSIM (Feature Similarity Index) for local feature matching accuracy.

First, the similarity metrics were computed between the expert group (e) and the novice group (n) in relation to the template group (s). Table 3 presents the descriptive statistics for all metrics. For the PSNR, the expert group had Mean = 32.19 and SD = 2.02, while the novice group had Mean = 32.28 and SD = 2.07. The mean difference between the two groups was minimal, and *t*-test results indicated no significant difference (*t* = −0.23, *p* = 0.81). For the SSIM, the expert group had Mean = 0.79 and SD = 0.06, and the novice group had Mean= 0.79 and SD = 0.07. Again, the difference was negligible, with no significant difference observed in the *t*-test (*t* = −0.19, *p* = 0.85). For the FSIM, both the expert and novice groups had Mean = 0.35 and SD = 0.03, with *t* = 0.07 and *p* = 0.95. These results indicate high structural consistency between the expert and novice groups in terms of morphological similarity, demonstrating that the tracing method effectively reduced individual differences between writers. Consequently, the traced results of experts and novices were highly similar in overall form, with minimal variation in similarity metrics. This outcome establishes a solid basis for further comparisons involving calligraphic style analysis, confirming that the influence of writer expertise on static structure has been effectively controlled.

**Figure 6:**
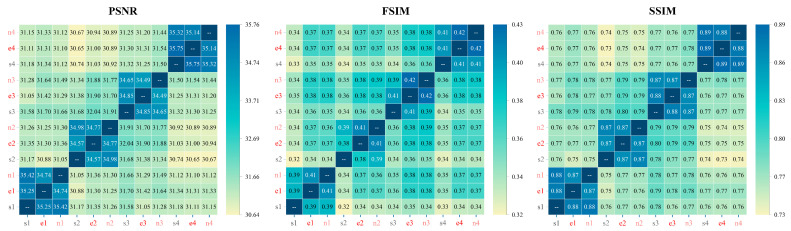
Similarity metrics (PSNR, FSIM, SSIM) for different calligraphy samples by writer and font style. This figure shows the similarity calculations between different writer groups (e, n, s) across four font styles (id = 1, 2, 3, 4). The color gradient represents similarity, with darker colors indicating higher similarity and lighter colors indicating lower similarity. The heatmaps show that the tracing method control effectively reduces individual style differences, resulting in high similarity within the writers, while cross-font combinations show lower similarity.

After controlling for writer experience differences, we further introduced calligraphic style (id = 1, 2, 3, 4) as an additional variable, in addition to the writer variable (writer = e, n, s), to perform grouped similarity computations. Figure 6 presents the results of three similarity metrics across different character combinations, where the *x* axis and *y* axis represent sample pairs (e.g., the cell e1-n1 indicates the similarity score between the expert and novice traced copies of calligraphic style 1). The numerical values and color gradients reflect similarity levels (PSNR: 30.65–35.75, FSIM: 0.32–0.42, SSIM: 0.73–0.89), where darker colors indicate lower similarity, and lighter colors indicate higher similarity.

The heatmap analysis reveals two key patterns:

1. High similarity along the diagonal: All three similarity metrics exhibit a distinct 3 × 3 block pattern along the diagonal (bottom-left to top-right), where the 3 × 3 matrix cells along the diagonal show notably high similarity. This suggests that different writers’ traced results for the same calligraphic style exhibit significantly high structural similarity (e.g., e1-s1 and e1-n1). This finding confirms that the tracing method effectively constrained writers to adhere to the structural form of the template, thereby suppressing the influence of individual stylistic variations to some extent.
2. Lower similarity in cross-style comparisons: The similarity scores for cross-style combinations are substantially lower than those for same style, different writer combinations. For example, within the expert group, the cross-style similarity (e1-e2: PSNR = 31.30, FSIM = 0.37, SSIM = 0.77) is significantly lower than the same style, cross-writer similarity (e1-n1: PSNR = 34.74, FSIM = 0.41, SSIM = 0.87). As previously discussed, all four calligraphic styles belong to Kaishu (regular script) and inherently exhibit a high degree of similarity. However, the heatmap reveals a notable trend—the similarity between different writers is higher than the similarity between different calligraphic styles.

**Figure 7:**
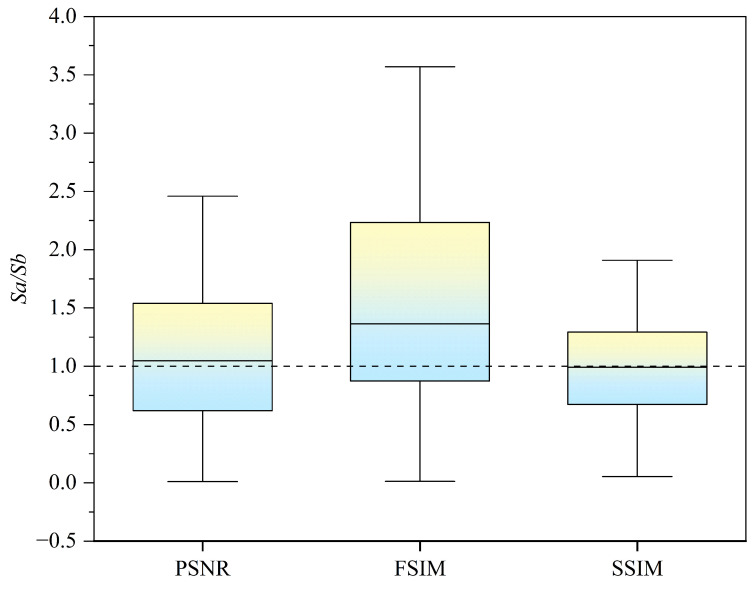
Calculate the similarity between two of the three writing results (e, n, s) as *Sa*. Calculate the similarity between two of the four styles of writing (ID) as *Sb*. Construct the value of the distribution of the Sa/Sb ratio is shown in this figure. From the three kinds of similarity results, most of the ratios are more than 1, which indicates that the similarity between the writers is higher than the similarity between the styles of writing.

To quantify the relative relationship between inter-writer similarity and inter-style similarity and further confirm the high similarity of traced results among different writers, this study constructed the similarity ratio Sa/Sb as a feature value for analysis. Here, *Sa* represents the similarity between different writers’ traced results, while *Sb* represents the similarity between different calligraphic styles. If Sa/Sb > 1, it indicates that the similarity between different writers in the traced task is higher than the similarity between different calligraphic styles. The results, presented in Figure 7, show that the Sa/Sb values are significantly greater than 1 in most samples, further supporting the hypothesis that traced results among different writers exhibit high similarity. This finding provides statistical evidence for the controllability of static features, demonstrating that the model tracing control method effectively suppressed individual stylistic variations in morphology.

This section conducted a stepwise analysis to examine static morphological differences in traced results. First, descriptive statistical results indicated that calligraphy experts and novices exhibited extremely high similarity in static morphology. Second, a comparative analysis of inter-writer similarity and inter-style similarity revealed that the variation in similarity between different writers was smaller than the variation between different calligraphic styles. Finally, the constructed similarity ratio Sa/Sb was used for quantitative validation of this conclusion. These findings demonstrate that through a standardized tracing process, the traced results of calligraphy experts and novices exhibited high similarity. In summary, within the aesthetic context of calligraphic tracing examined in this study, the static morphological differences in traced results were not significant.

#### 3.2.2. Static Features Fail to Effectively Predict Aesthetic Evaluation

In the previous section, we confirmed that the morphological differences in traced results were effectively controlled. However, whether static features still influence aesthetic evaluation requires further investigation. Although the tracing method ensured a high degree of morphological similarity among different writers, the influence of static features is not limited to morphological similarity alone—it also includes stroke details, ink distribution, and other finer visual characteristics. Therefore, high morphological similarity does not necessarily imply that static features are irrelevant to aesthetic judgment. Based on this consideration, this section conducts a quantitative analysis of classical static features to evaluate their predictive power in calligraphic tracing aesthetic assessment, thereby providing stronger empirical evidence regarding the influence of static features in aesthetic judgment.

This study extracted 26 interpretable classical static features based on the morphological structure, ink distribution, and other artistic characteristics of calligraphy fonts, as detailed in Table 4. These features encompass key visual elements of calligraphic works, including character complexity, ink usage, and contour characteristics. Moreover, the selected static features have been widely validated in previous calligraphic aesthetic research Brachmann and Redies (2017); M. Li et al. (2019); Sun et al. (2009); Xu et al. (2018); P. Zhou et al. (2021). We aim to examine the ability of these classical static features to predict aesthetic evaluation in traced results. To achieve this, we employed two modeling approaches: multiple linear regression (MLR) and multilayer perceptron (MLP). These methods were applied in a progressive manner to systematically validate the actual role of static features in aesthetic assessment.

The multiple linear regression (MLR) method was employed to evaluate the linear relationship between classical static features and aesthetic ratings. Specifically, the regression model was constructed by setting each aesthetic rating dimension (strength, fluency, beauty, and skill) as the dependent variable, while the static features (*f1*~*f26*) served as independent variables. The model was fitted to calculate the correlation between each feature and the corresponding rating dimension. By analyzing regression coefficients and *R*^2^, we assessed the explanatory power and contribution of each static feature in predicting aesthetic ratings. As shown in Table 5, the *R*^2^ values were generally low across all four rating dimensions (strength *R*^2^ = 0.148, fluency *R*^2^ = 0.070, beauty *R*^2^ = 0.063, skill *R*^2^ = 0.062). These results indicate that static features have limited explanatory power in capturing the variance of aesthetic ratings, suggesting that they are insufficient to effectively model the complexity of each rating dimension. This finding preliminarily validates the limitations of static features in the aesthetic evaluation of calligraphic tracing in this study.

To comprehensively evaluate the predictive effectiveness of static features in calligraphic tracing aesthetics, we further introduced neural network modeling to address the limitations of multiple linear regression (MLR) in handling nonlinear relationships. Neural networks can capture potential nonlinear patterns between multiple static features and aesthetic ratings, thereby improving predictive performance.

The multilayer perceptron (MLP) model, a commonly used deep learning model, was employed in this study. MLP utilizes multiple hidden layers and nonlinear activation functions to learn complex relationships between input features and target variables. Here, we constructed separate MLP models for each of the four rating dimensions, with the input layer consisting of 26 standardized classical static features and the output layer generating a single regression value for the corresponding rating dimension. The model adopted a fully connected multi-layer structure, incorporating the ReLU activation function to enhance nonlinear fitting capabilities. To improve generalization ability, Dropout layers were applied to randomly deactivate a subset of neurons, while L2 regularization was imposed on all weights to prevent overfitting. During training, we employed the Adam optimizer for weight updates, along with ReduceLROnPlateau for dynamic learning rate adjustments, enhancing convergence efficiency. Additionally, we used manual grid search to optimize five key hyperparameters, including the number of hidden layers (1, 2, 3), the number of neurons per layer (32, 64, 128), Dropout rates (0.2, 0.3, 0.5), learning rates (1 × 10^−3^, 1 × 10^−4^, 1 × 10^−5^), and L2 regularization coefficients (0.01, 0.001, 0.0001). For each rating dimension, we iterated over 243 hyperparameter combinations, trained the model on the training set, and evaluated Mean Squared Error (MSE) on the validation set. The best hyperparameter configuration, determined by the lowest validation loss, was then applied to the test set to assess model performance.

As shown in Table 6, despite extensive neural network optimizations, the model’s predictive capability for aesthetic evaluation remained limited. For the fluency dimension, MSE = 1.735 and MAE = 1.083, with *R*^2^ = −0.032, indicating that the neural network failed to capture rating trends effectively. The negative *R*^2^ value suggests that the model performed poorly in predicting fluency ratings. For the other dimensions, the *R*^2^ values were positive but generally low (strength *R*^2^ = 0.029, beauty *R*^2^ = 0.008, skill *R*^2^ = 0.080), demonstrating that predictive performance across all rating dimensions remained weak.

**Figure 8:**
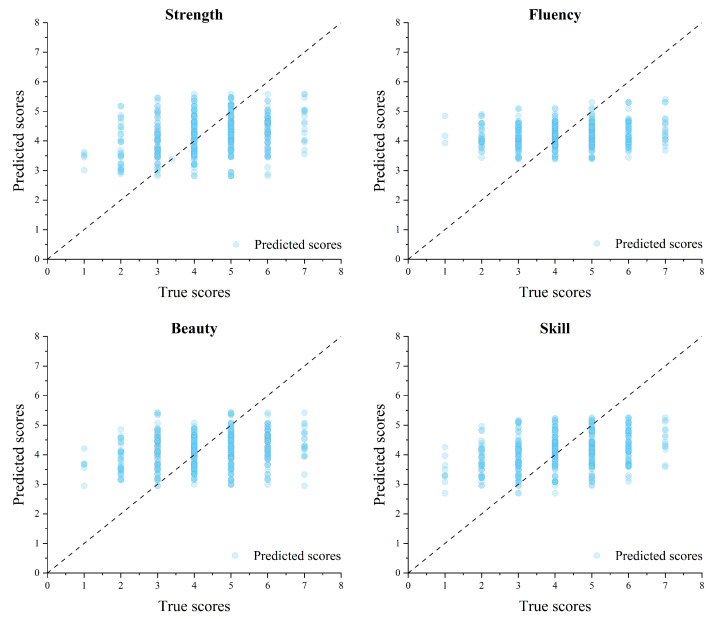
This is a scatter plots of True vs. Predicted Scores for strength, fluency, beauty, and skill. As can be seen from the chart, the model has poor predictive ability for the four dimensions of aesthetic evaluation.

As shown in Figure 8, we plotted scatter diagrams comparing true scores (actual ratings) and predicted scores (model outputs) for each aesthetic rating dimension. The dotted line in the figure represents the ideal diagonal line, where predicted values perfectly match actual values. It is evident that the scatter distribution exhibits significant dispersion across all rating dimensions, indicating that the model’s fitting performance was poor for every dimension. These findings suggest that the MLP model based on classical static features has substantial limitations in predicting aesthetic evaluation. Furthermore, this result confirms that even with nonlinear modeling approaches, classical static features fail to effectively predict aesthetic judgments in calligraphic tracing.

Through the above analysis, it is evident that whether using linear or nonlinear modeling approaches, relying solely on static features fails to accurately predict the aesthetic evaluation of calligraphic traced works. This finding demonstrates that static features have poor predictive performance in calligraphic tracing aesthetic assessment.

This study established a rigorous argumentation framework based on two key pieces of evidence. First, from the perspective of static morphological similarity, we validated that there were no significant differences in the static structure of traced results between different writers. Second, both linear and nonlinear predictive models were constructed, and the results show that classical static features exhibited poor predictive capability in aesthetic evaluation of calligraphic tracing. By integrating these two conclusions, we provide comprehensive evidence that static features play a weak role in calligraphic tracing aesthetic judgment, thereby confirming Hypothesis 2.

## 4. Discussion

### 4.1. Research Findings and Interpretation

The core question of this study is whether dynamic features play a dominant role over static features in the aesthetic judgment of calligraphic tracing. To explore this, we first examined the significant influence of dynamic features on aesthetic evaluation, thereby testing Hypothesis 1. The results of the aesthetic evaluation experiment revealed that aesthetic judgments varied significantly between different writers (see Table 2) and that presentation formats incorporating dynamic information, such as brushstroke trajectory video *b* or pen-holding writing video *f*, received significantly higher ratings in strength, fluency, beauty, and skill compared to the static result sequence *s*. One-way ANOVA further confirmed this difference, showing that presentation format had a highly significant effect on aesthetic ratings and that the inclusion of dynamic information significantly enhanced viewers’ aesthetic evaluation of the works (see Figure 5). This effect was observed in both expert and novice viewers, with the most pronounced improvements in the fluency and strength dimensions. In summary, motion cues provided by dynamic features play a crucial role in aesthetic judgment, supporting Hypothesis 1.

The visual imagination theory proposed ingby Walton (1993) provides insight into the role of dynamic features in aesthetic judgment. When appreciating calligraphic works, viewers may mentally simulate the movement of brushstrokes, forming motor imagery that enhances emotional engagement with the artwork. This simulation process enables viewers to better perceive the rhythm and expressive strength of brushstrokes, thereby enhancing their aesthetic experience. Previous studies by Freedberg et al. also support this mechanism, demonstrating that unconscious motor simulation contributes to enhanced aesthetic appreciation in the context of painting evaluation Freedberg and Gallese (2007). These findings align with the results of this study, indicating that dynamic features play a key role in aesthetic judgment by evoking motor resonance. Additionally, we found that the aesthetic ratings of static traced results differed significantly between experts and novices across all evaluation dimensions. We hypothesized that these differences are primarily driven by implicit dynamic features rather than static features, a hypothesis that we further validated in subsequent analyses.

To validate the above hypothesis, we assessed the weak influence of static features on aesthetic judgment from two perspectives, thereby testing Hypothesis 2. First, we verified that the tracing method effectively eliminated static morphological differences between different writers. Multiple similarity metrics (PSNR, SSIM, and FSIM) were employed to quantify the similarity between traced results and the template character (see Table 3), and the results indicated minimal differences in static morphological structure. This suggests that, in the calligraphic tracing context of this study, observers did not primarily rely on static features in their aesthetic judgments but were instead more influenced by implicit dynamic features. Building on this, we further examined the predictive effectiveness of classical static features in aesthetic evaluation of calligraphic tracing and found that these features, despite being validated in prior aesthetic studies, failed to accurately predict aesthetic ratings in this study. Multiple linear regression analysis showed low *R*^2^ values across all aesthetic dimensions (see Table 5), indicating that static feature-based linear regression models performed poorly in predicting the aesthetic evaluation of calligraphic tracing. Even after introducing a nonlinear multilayer perceptron (MLP) model, the predictions generated by the optimal parameter configuration still deviated significantly from actual scores, with poor model fitting performance (see Table 6 and Figure 8). The results from both analytical approaches confirm the limited role of static features in calligraphic tracing aesthetic assessment, thereby supporting Hypothesis 2.

It should be emphasized that the 26 features used in modeling were intentionally selected based on previous quantitative aesthetic research, where they had been shown to be significantly effective Brachmann and Redies (2017); M. Li et al. (2019); Sun et al. (2009); Xu et al. (2018); P. Zhou et al. (2021). However, our results show that these features fail to predict the aesthetic evaluation of traced works effectively. We argue that this outcome does not invalidate the value of static features, but rather highlights the particularity of the calligraphic tracing aesthetic context. In our experiment, all stimuli were generated by tracing the same standard reference character, resulting in highly similar morphological structures. Under such conditions, traditional static features focused on form and structure performed poorly. The differences in aesthetic ratings are more likely driven by implicit dynamic features capable of evoking motor imagery, such as stroke order, brush movement, variation in pressure, and even the writer’s expressive intent W. Chen et al. (2017). Therefore, relying solely on static feature modeling leads to limited predictive power, and it becomes essential to account for the role of implicit dynamic features. This finding further substantiates our central claim: in the aesthetic context of calligraphic tracing, the aesthetic judgment process relies more heavily on implicit dynamic features than on the information conveyed by static features alone.

In conclusion, the experimental results of this study validate the dominant role of dynamic features in the aesthetic judgment of calligraphic tracing while also demonstrating the relatively weak influence of static features.

### 4.2. Establishing a Dynamically Adaptive Aesthetic Model

Existing classical aesthetic cognition models face limitations in explaining the findings revealed in our experiment. Take, for example, Leder et al.’s aesthetic information processing model, which divides aesthetic processing into five stages: perception, unconscious classification, conscious style/content classification, knowledge-based cognitive processing, and final integrative evaluation Leder et al. (2004). These models emphasize a stepwise analysis and accumulation of information by the observer, focusing primarily on static features of the artwork (e.g., visual form, content semantics, etc.) and the observer’s prior knowledge. However, they lack a sufficient explanation of the role of dynamic features in the aesthetic process.

Our experimental findings, in contrast, demonstrate that dynamic features play an indispensable role in aesthetic evaluation. While neuroaesthetics research has touched upon dynamic features, such as Gallese et al.’s mirror neuron theory, which suggests that action simulation enhances aesthetic empathy Freedberg and Gallese (2007), these models typically treat dynamic features as secondary mechanisms within static perception, without clearly defining their dominant role in different contexts. Similarly, in the VIMAP model proposed by Yang et al., dynamic information is incorporated through “movement accessibility” in neural activation patterns Yang et al. (2019). However, this framework remains centered on static content perception and does not explicitly address the interaction between dynamic and static features. In summary, traditional aesthetic models exhibit clear deficiencies in explaining the role of dynamic features, highlighting the urgent need for a new framework that integrates both static and dynamic factors to provide a more comprehensive explanation of aesthetic judgment mechanisms.

**Figure 9:**
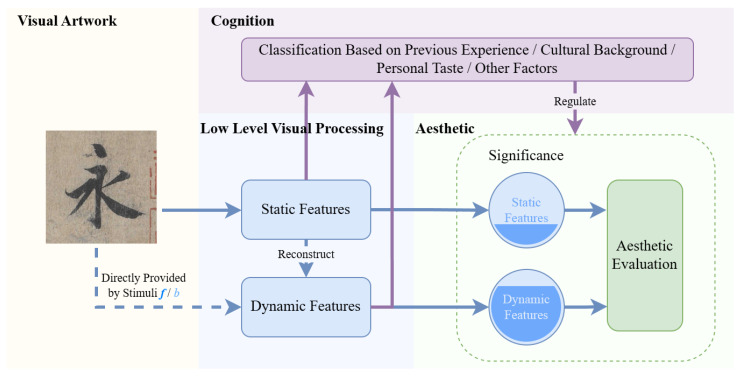
This is the aesthetic cognition model proposed in this study, which describes the process of aesthetic evaluation. In this process, the viewer first undergoes low-level visual processing, decomposing the artwork into static and dynamic features. At the cognitive level, the weights of these features are adjusted based on the aesthetic context, ultimately influencing aesthetic judgment.

To address these limitations, we propose a new framework for the aesthetic mechanism model, as illustrated in Figure 9, which incorporates dynamic features into the model and introduces a mechanism for dynamically adjusting the weighting of static and dynamic features based on the aesthetic context.

In this aesthetic model, the aesthetic process is decomposed as follows: When an observer engages with a visual artwork, the visual input first undergoes basic perceptual processing, where it is parsed into static and dynamic features. Next, the observer classifies the aesthetic context based on personal prior experience, cultural background, and individual preferences and adjusts the relative weight of static and dynamic features accordingly. For instance, in the aesthetic context of calligraphic tracing, the observer simulates the motor process of tracing, leading to a significant increase in the weighting of dynamic features, which dominates aesthetic judgment. In contrast, in the aesthetic context of photography, the weighting shifts toward static features such as color contrast and compositional balance, which become the primary basis for evaluation. Finally, the aesthetic judgment output is generated based on the adjusted relative importance of static and dynamic features.

This context-dependent weight adjustment mechanism helps to provide a deeper understanding of the flexible and multilayered psychological processes observed in aesthetic experience. Additionally, it offers new insights and a more comprehensive theoretical foundation for future research on aesthetic models. Furthermore, the interaction between dynamic and static features enables a more holistic understanding of complex aesthetic cognition processes, ultimately leading to a better explanation of aesthetic preferences.

### 4.3. Limitations and Future Directions

Although this study has yielded meaningful conclusions, there are still several limitations that should be considered. First, this study focused exclusively on calligraphy as an art form, and the role of dynamic features in other artistic domains remains unclear. Future research could extend the methodology of this study to other visual art disciplines to explore the impact of implicit dynamic features on aesthetic judgment across different art forms. By incorporating dynamic features into aesthetic evaluation experiments in broader artistic contexts, researchers can examine the generalizability of dynamic feature effects and further enrich theoretical models related to aesthetic cognition mechanisms.

Second, while this study highlights the significant role of dynamic features in the aesthetic process, it does not provide a detailed interpretability framework for these features. Future studies could integrate more advanced motion capture and writing recording technologies to acquire higher-resolution dynamic process data and extract a richer set of dynamic feature indicators. This approach would help identify which specific dynamic elements have the greatest impact on aesthetic evaluation, thereby offering deeper insights into the mechanisms through which dynamic features influence aesthetic judgment.

Finally, the current experimental design may be subject to potential influences from emotional appeal and differences in information quality. In particular, the dynamic presentation conditions (*b and f*) may have been more visually engaging or provided clearer informational cues, which could introduce confounding effects on aesthetic ratings. It is important to acknowledge that the static condition (*s*) lacks temporal cues and indicators of movement, which may limit observers’ ability to fully assess the quality of the work. This could lead to more conservative or uncertain ratings. Future studies should further refine experimental controls, aiming to systematically balance the amount of information, perceptual modalities, and levels of emotional arousal across different presentation formats. Such improvements would help to more accurately isolate the true contribution of dynamic features to aesthetic judgment and minimize interference from other potential variables.

In conclusion, future research should continue to focus on the dynamic dimensions of artistic creation, leveraging emerging technologies and interdisciplinary approaches to further expand and deepen our understanding of the principles underlying aesthetic judgment in art.

## 5. Conclusions

Through rigorous experimental design and analysis, this study systematically validated the positive influence of dynamic features on aesthetic judgment in calligraphic tracing (Hypothesis 1) and demonstrated that static features exerted a relatively weaker influence in this context (Hypothesis 2). Based on these findings, we conclude that dynamic features dominate aesthetic judgment in the context of calligraphic tracing. This conclusion strongly supports the crucial role of dynamic features in aesthetic evaluation, providing a new perspective for artistic aesthetic research and highlighting the previously overlooked significance of dynamic features in aesthetic judgment. Based on these results, we propose a hypothetical aesthetic cognition model that integrates static and dynamic features, outlining a dynamic weighting adjustment mechanism between the two feature types depending on the specific aesthetic context. This model offers new insights into the complex processes underlying aesthetic perception.

In conclusion, this study makes a unique contribution to the field of aesthetic psychology, demonstrating that in certain artistic contexts, dynamic features can surpass static features to become the dominant factor in aesthetic judgment. We encourage future research in artistic aesthetics to continue exploring the combined influence of static and dynamic features on viewing experiences, providing deeper insights into the sources of aesthetic value in artworks and offering valuable implications for art education and creative practice.

## Figures and Tables

**Table 1:** This table presents the selected calligraphy samples (s), which were categorized based on three dimensions: character structure (six distinct structural types), complexity (high, low), and calligraphic style (Yan Zhenqing, Liu Gongquan, Ouyang Xiu, Zhao Mengfu). Additionally, the table includes the corresponding traced results produced by the calligraphy expert (e) and the novice (n).

Structure	Complexity	Yan Zhenqing			Liu Gongquan			Ouyang Xiu			Zhao Mengfu		
		s	e	n	s	e	n	s	e	n	s	e	n
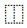	low	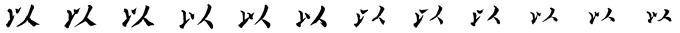											
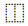	High	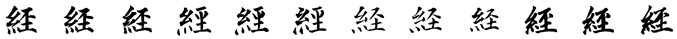											
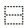	Low	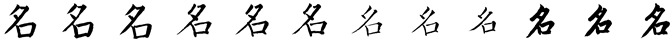											
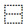	High	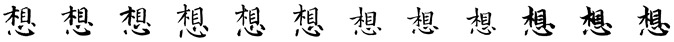											
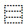	Low	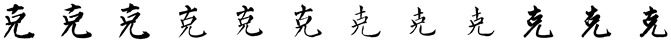											
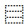	High	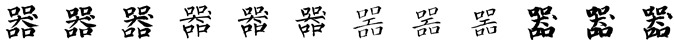											
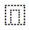	Low	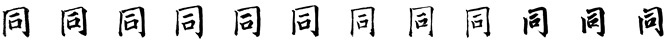											
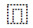	High	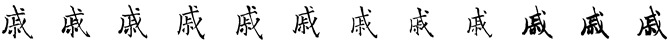											
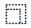	Low	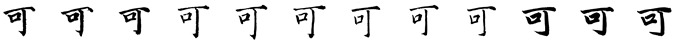											
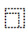	High	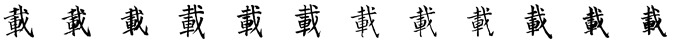											
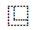	Low	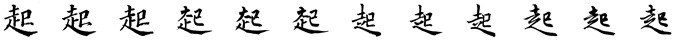											
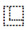	High	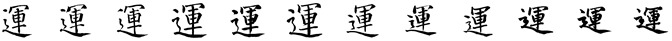											

**Table 2:** Descriptive statistics of calligraphy evaluation scores. The results show that the ratings of calligraphic works produced by experts are significantly higher than those of produced by novices. In addition, the ratings for expert-produced works are more stable, while those for novice-produced works exhibit greater fluctuations.

Writer	Method	Statistics	Strength	Fluency	Beauty	Skill
e	*s*	Mean	4.49	4.56	4.58	4.52
		Std. Deviation	1.32	1.33	1.38	1.39
		Grouped Median	4.56	4.64	4.66	4.62
	*f*	Mean	4.68	4.93	4.69	4.64
		Std. Deviation	1.19	1.24	1.25	1.31
		Grouped Median	4.69	5.00	4.74	4.69
	*b*	Mean	4.52	4.82	4.62	4.69
		Std. Deviation	1.28	1.19	1.29	1.30
		Grouped Median	4.52	4.83	4.68	4.77
	Total	Mean	4.56	4.77	4.63	4.61
		Std. Deviation	1.26	1.27	1.31	1.34
		Grouped Median	4.59	4.84	4.70	4.72
n	*s*	Mean	4.08	4.10	3.96	3.83
		Std. Deviation	1.33	1.33	1.40	1.40
		Grouped Median	4.07	4.10	3.96	3.79
	*f*	Mean	4.33	4.24	4.20	4.13
		Std. Deviation	1.23	1.24	1.26	1.31
		Grouped Median	4.39	4.27	4.22	4.19
	*b*	Mean	4.15	4.23	4.23	4.14
		Std. Deviation	1.26	1.29	1.38	1.45
		Grouped Median	4.17	4.25	4.30	4.23
	Total	Mean	4.19	4.19	4.12	4.03
		Std. Deviation	1.28	1.29	1.35	1.39
		Grouped Median	4.21	4.21	4.15	4.07

**Table 3:** This table shows the descriptive statistics and *t*-test results for similarity metrics between expert and novice.The *t*-test results show no significant differences, indicating that the expert and novice groups have a high degree of consistency in static features.

Metric	Mean ± SD		*t*	*p*	*df*
	Expert	Novice			
PSNR	32.19±2.02	32.28±2.07	−0.235	0.815	131
SSIM	0.79±0.06	0.79±0.07	−0.190	0.850	131
FSIM	0.35±0.03	0.35±0.03	0.067	0.946	131

**Table 4:** Definitions of selected classical static features.

Feature	Definitions
*f1*	Perimetric Complexity. A measure of the image’s boundary complexity calculated by analyzing the perimeter and ink area based on the Pelli algorithm.
*f2*	Space Utilization. It describes the relationship between ink usage and the space occupied by the font.
*f3*	Aspect Ratio. The ratio of the length to the width of the character’s minimum bounding rectangle.
*f4*	Rectangularity. Describes the closeness of the character to a rectangle and is calculated as the ratio of the character’s convex hull perimeter to the perimeter of its minimum bounding rectangle.
*f5*	Roundness. Measures the closeness of the character’s convex hull to a circle.
*f6*	Eccentricity. It is used to calculate the eccentricity of the character’s minimum bounding rectangle.
*f7*, *f8*	The inclination angle of the fitted line and slope. Describes the character’s tilt degree.
*f9*~*f12*	Pixel Projection Variances. These metrics capture the stroke distribution by calculating the variances of vertical projections at 0°, 45°, 90°, and 135° angles.
*f13*~*f16*	Ink Distribution. Calculates the convex hull area to ink ratio for the four quadrants with the center of the convex hull as the origin.
*f17*	Quadrant Pixel Distribution Ratio Variance. It is the variance of f13 f16.
*f18*	Fitted Line Segmentation Area Ratio. The fitted line divides the convex hull into two regions, calculating the ratio between the smaller area and the larger area.
*f19, f20*	Centroid X, Centroid Y. The coordinates of the contour’s center, which is calculated as the average position of all points in the x and y directions based on the image moments.
*f21*	Central Area. The ratio of black pixels in the center grid to the total black pixels, with the character’s bounding box divided into a 3 × 3 grid.
*f22*	Contour Entropy. Using Freeman encoding, we traverse the font’s contour to generate a sequence of relative pixel positions and calculate its entropy.
*f23*	Enclosed Space Count. The number of enclosed regions in a character.
*f24*	Enclosed area proportion. The ratio of enclosed regions’ area to the total character space.
*f25*	Number of Endpoints. The number of endpoints in the character.
*f26*	Number of Intersections. The number of stroke intersections in the character.

**Table 5:** This table presents the results of the multiple linear regression analysis, examining the relationship between static features and aesthetic ratings across four dimensions: strength, fluency, beauty, and skill. The analysis suggests that static features have a weak correlation with aesthetic ratings and provide limited explanatory power for the scores.

Model	*R* ^2^	Adjusted *R*^2^	SEE	Pearson Correlation
Strength	0.148	0.139	1.246	0.385
Fluency	0.070	0.059	1.306	0.265
Beauty	0.063	0.052	1.383	0.250
Skill	0.062	0.052	1.398	0.250

SEE: Std. error of the estimate.

**Table 6:** This table presents the performance of neural network model in predicting aesthetic ratings: MSE, MAE, *R*^2^, and Pearson Correlation for four dimensions. These indicators show that the model using classical static feature modeling does not perform well in predicting calligraphy evaluations.

Model	MSE	MAE	*R* ^2^	Pearson Correlation
Strength	1.665	1.054	0.029	0.296
Fluency	1.735	1.083	−0.032	0.124
Beauty	1.804	1.090	0.008	0.236
Skill	1.712	1.072	0.080	0.318

## Data Availability

The original contributions presented in this study are included in the article. Further inquiries can be directed to the corresponding author.
